# PARP Inhibition Sensitizes to Low Dose-Rate Radiation TMPRSS2-ERG Fusion Gene-Expressing and PTEN-Deficient Prostate Cancer Cells

**DOI:** 10.1371/journal.pone.0060408

**Published:** 2013-04-02

**Authors:** Payel Chatterjee, Gaurav S. Choudhary, Arishya Sharma, Kamini Singh, Warren D. Heston, Jay Ciezki, Eric A. Klein, Alexandru Almasan

**Affiliations:** 1 Department of Cancer Biology, Lerner Research Institute, Cleveland Clinic, Cleveland, Ohio, United States of America; 2 Department of Radiation Oncology, Taussig Cancer Institute, Cleveland Clinic, Cleveland, Ohio, United States of America; 3 Glickman Urological and Kidney Institute, Cleveland Clinic, Cleveland, Ohio, United States of America; 4 Kent State University, Kent, Ohio, United State of America; 5 Department of Pathology, Case Western Reserve University, Cleveland, Ohio, United States of America; 6 Cleveland State University, Cleveland, Ohio, United States of America; National Cancer Institute, United States of America

## Abstract

Exposure to genotoxic agents, such as irradiation produces DNA damage, the toxicity of which is augmented when the DNA repair is impaired. Poly (ADP-ribose) polymerase (PARP) inhibitors were found to be “*synthetic lethal*” in cells deficient in BRCA1 and BRCA2 that impair homologous recombination. However, since many tumors, including prostate cancer (PCa) rarely have on such mutations, there is considerable interest in finding alternative determinants of PARP inhibitor sensitivity. We evaluated the effectiveness of radiation in combination with the PARP inhibitor, rucaparib in PCa cells. The combination index for clonogenic survival following radiation and rucaparib treatments revealed synergistic interactions in a panel of PCa cell lines, being strongest for LNCaP and VCaP cells that express ETS gene fusion proteins. These findings correlated with synergistic interactions for senescence activation, as indicated by β--galactosidase staining. Absence of PTEN and presence of ETS gene fusion thus facilitated activation of senescence, which contributed to decreased clonogenic survival. Increased radiosensitivity in the presence of rucaparib was associated with persistent DNA breaks, as determined by χ-H2AX, p53BP1, and Rad51 foci. VCaP cells, which harbor the *TMPRSS2-ERG* gene fusion and PC3 cells that stably express a similar construct (fusion III) showed enhanced sensitivity towards rucaparib, which, in turn, increased the radiation response to a similar extent as the DNA-PKcs inhibitor NU7441. Rucaparib radiosensitized PCa cells, with a clear benefit of low dose-rate radiation (LDR) administered over a longer period of time that caused enhanced DNA damage. LDR mimicking brachytherapy, which is used successfully in the clinic, was most effective when combined with rucaparib by inducing persistent DNA damage and senescence, leading to decreased clonogenic survival. This combination was most effective in the presence of the *TMPRSS2-ERG* and in the absence of *PTEN*, indicating clinical potential for brachytherapy in patients with intermediate and high risk PCa.

## Introduction

Prostate cancer (PCa) is the most frequently diagnosed tumor in men, accounting alone for 29% of incident cases [1]. It is the second most common cause of death due to cancer in men after lung cancer. Irradiation is an important treatment modality for PCa, with a clinical response achieved of ∼85%. It is used primarily for early-stage disease, as an adjuvant to surgery, and in combination with chemo-therapeutics that allow its use at lower doses, with higher efficiencies and with less cytotoxic effect to the adjacent normal tissues.

Poly (ADP-ribose) polymerase (PARP) is represented by a family of proteins that are expressed abundantly, are primarily localized in the nucleus, and are involved in many important cellular processes, such as the response to DNA damage, its repair, and when the damage is severe, cell death through apoptosis or necrosis [2]. PARP-1 and -2 are known to have a role in various DNA repair mechanisms, such as base excision repair, homologous recombination (HR) [3], and nonhomologous end-joining (NHEJ) [4]. After detecting DNA damage, with the help of its DNA-binding domain, activated PARP-1 triggers poly-ADP ribosylation of histones and PARP-1 itself. Importantly, PARP-1 ensures regulation of DNA replication fork progression by HR on damaged DNA [5]. PARP-1 is involved mainly in the repair of single-stranded breaks, which, if unrepaired are converted to double-stranded breaks (DSBs) during DNA replication. PARP inhibitors (PARPi) represent a new class of agents that prevent the synthesis of poly-ADP ribose by impinging on the downstream DNA repair processes, and as a result the DNA damage persists [2]. PARPi, therefore, can act as a single agent in HR-deficient tumors (e.g. BRCA1/2-defective) through “*synthetic lethality*” [6]. Disabling NHEJ in HR-deficient cells via DNA-PKcs inhibition can reverse the effect of PARPi-mediated lethality [7]. Radiation induces PARP activity and its inhibition enhances cell death and improves tumor growth delay in irradiated lung cancer models [8]. Levels of PARP-1 are also increased in advanced, castrate resistant PCa [9], [10], therefore, its use in combination with radiation to enhance radiotherapy is appealing.

The “*synthetic lethality*” concept has been effective in mammalian cells in tumor models with defective BRCA1 or BRCA2, which, as a consequence have defective HR [3] and are thus amenable to use of PARPi as a monotherapy [11]. However, for tumors where such mutations are rare, such as PCa, there is an urgent need to identify additional DNA damage and repair defects that can provide a “*synthetic lethal*” combination with PARPi. Thus, extending the use of PARPi beyond tumors with defective BRCA1/2 is of great interest.

In late stage PCa, bi-allelic deletion of the *PTEN* (Phosphatase and tensin homolog) gene is a common occurrence that has been suggested to impact HR DNA repair. PTEN antagonizes the PI3K/AKT survival pathway by its phospholipid 3-phosphatase activity, which, in turn, regulates proliferation, migration, and apoptosis. Complete loss of *PTEN* also stimulates a strong senescence response that acts as an additional mechanism for tumor suppression. Senescence has been proposed to function as an anti-tumor mechanism in response to DNA damage by inducing an irreversible growth arrest and restricting the replicative life span of cells [12]. Similar to BRCA1/2-defective tumor cells, *PTEN*-null PCa cells have been reported to be sensitive to PARPi.

In this study, we examined the response to a potent PARP inhibitor, rucaparib alone or in combination with radiation in a panel of PCa cell lines. Our data support the effectiveness of rucaparib as a potent PARPi for radiosensitizing PCa cells, most effectively when used at low dose-rates in cells that harbor the *TMPRSS2-ERG* gene fusion or are *PTEN*-deficient.

## Materials and Methods

### Cell Culture

Human PCa cell lines PC3, LNCaP, DU145, and VCaP were obtained from ATCC (Manassas, VA); C4-2 was described earlier [13]. Cells were cultured in RPMI-1640 medium, except DU145 (DMEM) and VCaP (modified DMEM) supplemented with 10% fetal bovine serum (Atlanta Biologicals), L-glutamine (Invitrogen), and 100 unit/ml penicillin-streptomycin (Invitrogen) in a humidified incubator at 37°C and 5% CO_2_. Stock solutions of rucaparib, provided by Pfizer, were made in DMSO (Sigma Aldrich).

For transfection, cells were seeded at ∼80% confluency in an antibiotic-free media. *TMPRSS2-ERG* fusion III (the most common) isoform [14] generously provided by Dr. Michael Ittmann was transfected using lipofectamine 2000, followed by selection for neomycin resistance with 1 mg/ml G418 (Invitrogen). The efficiency of transfection was verified by Western blotting (Fig. S1A).

### Radiation Treatment

Ionizing radiation was delivered using a conventional cesium-137 χ-irradiator (JL Shepherd Associates, San Fernando, CA), at a dose rate of 146 cGy/min [15]. Dose-rate experiments were performed by changing the position of the plates or with the use of an attenuator. An Ir-192 source of radiation, which emits β-particles, employed a custom-fabricated cell irradiator, with the design of the device as described [16].

### Assays for Colony Formation and Senescence

For the colony formation assay, 500 cells/60-mm dish (or 750 cells/60-mm dish for LNCaP) were plated the day before treatment. Rucaparib was administered at the indicated doses continuously. Two weeks after treatment with radiation or/and rucaparib, cells were stained with 0.1% crystal violet, and cell colonies with >50 cells were scored by an alpha image analyzer (Alpha Innotech Corp). The senescence assay was performed as described [17]. After six or twelve days, cells were fixed and the percentage of β-galactosidase-positive cells was determined by counting ≥five different fields (∼70 cells/sample).

### Immunofluorescence

Cells were plated on coverslips in 35-mm culture dishes. After treatment, cells were fixed with 2.0% paraformaldehyde for 20 min at room temperature, washed 3× for 5 min with phosphate-buffered saline (PBS), permeabilized with 0.2% Triton X-100 in PBS for 10 min, and blocked in 3% FBS in PBS containing 0.1% Triton X-100 for 1 h. The coverslips were then immunostained using the antibodies against χ-H2AX (Millipore), 53BP1 (Abcam), or Rad51 (Santa Cruz Biotechnology), followed by a fluorescently-conjugated (Invitrogen) secondary antibody, as described [17]. Quantification was based on data observed from ≥70 cells.

### Statistical Analyses

For synergy analysis, cells were treated with rucaparib and irradiation, alone or in combinations in a ratio equaling the ratio of their median-effect doses, with each dose in each experiment plated in triplicate and each experiment performed three times. The interaction between the two treatments in clonogenic cell survival and senescence assays was then determined based on the isobolographic method of Chou and Talalay, as described earlier [18], [19]. All statistical analyses were done using two-way ANOVA and the statistical significance assigned for p<0.05.

### Western Blot Analyses

Cells were lysed and subjected to immunoblotting, as described [17], [20] and probed with antibodies against the V5 tag (Thermo Scientific), to detect the *TMPRSS2-ERG* fusion III gene and β-actin (Sigma Aldrich) as a loading control.

## Results

### Enhanced Sensitivity of PCa Cell Lines to Radiation when Combined with Rucaparib

**Figure 1:**
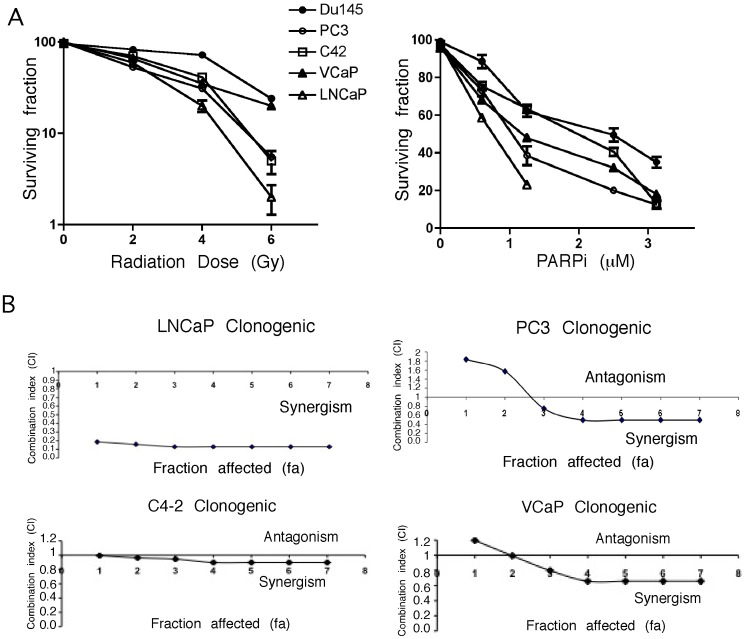
Decreased clonogenic survival of PCa cells exposed to radiation and rucaparib. **A**, Radiation and rucaparib dose response in PC3, C4-2, DU145, VCaP and LNCaP cells was established by clonogenic cell survival assays. Left panels indicate the response to radiation, those on the right to rucaparib. **B**, Synergistic effect of the combination of radiation and rucaparib on clonogenic survival in LNCaP, PC3, C4-2, and VCaP cells, where CI <1 represents synergy and CI >1 an antagonistic interaction between the two treatments. Error bars represent SD of mean (n = 3).

Ionizing radiation and DNA-damaging agents significantly induce PARP-1 and levels of PARP are higher in tumors [9], [10], therefore, PARPi could be used to sensitize to DNA-damaging chemo- or radio-therapy. Clinical success of PARPi on a cohort of patients [21] that included some with PCa prompted our interest in exploring the potential use of rucaparib (CO-338; formerly known as AG014699 and PF-01367338) as a radiosensitizer. Rucaparib, the first PARPi that has been developed [22], [23], and is currently tested in clinical trials has not been ––previously used for PCa cells. Examining its long-term effect on cell survival indicated a dose response for radiation and rucaparib for different PCa cells (Fig. 1A). VCaP and LNCaP (rucaparib concentration: 0.25, 0.5, and 0.75 µM) showed maximum sensitivity towards rucaparib, followed by PC3 and C4-2 cells. In combination with 1.5 Gy χ-irradiation, LNCaP cells exhibited the highest sensitivity to as low as 0.75 µM of rucaparib (Fig. 1B). Synergy calculations by isobologram analysis (see Materials and Methods) were performed for the four doses of radiation, ranging from 1–5 Gy in combination with rucaparib (concentration range 0.6–3.12 µM). For PC3, a concentration of rucaparib as low as 1.25 µM showed a significant decrease in colony number with a potent radiosensitization effect. DU145 cells were the least responsive to radiation and rucaparib, alone and in combination, with a limited effect obtained only at the highest doses. VCaP cells, however, while they showed a similar response to radiation as DU145, for the combination with rucaparib exhibited a synergistic interaction (Fig. 1B and Fig. S2A). The combination index revealed the strongest synergy (CI<0.2) in LNCaP cells following the radiation and rucaparib combination, with doses of radiation as low as 0.5 Gy and 0.25 µM of rucaparib being effective. PC3 cells exhibited a moderate synergy (CI = 0.7) following 4 Gy radiation and 2.5 µM of rucaparib, whereas C4-2 cells showed an additive effect (CI = 0.9) (Fig. 1B and Fig. S2).

### Rucaparib and Radiation Induce Senescence in PTEN-deficient and TMPRSS2-ERG Fusion-expressing Cells

**Figure 2:**
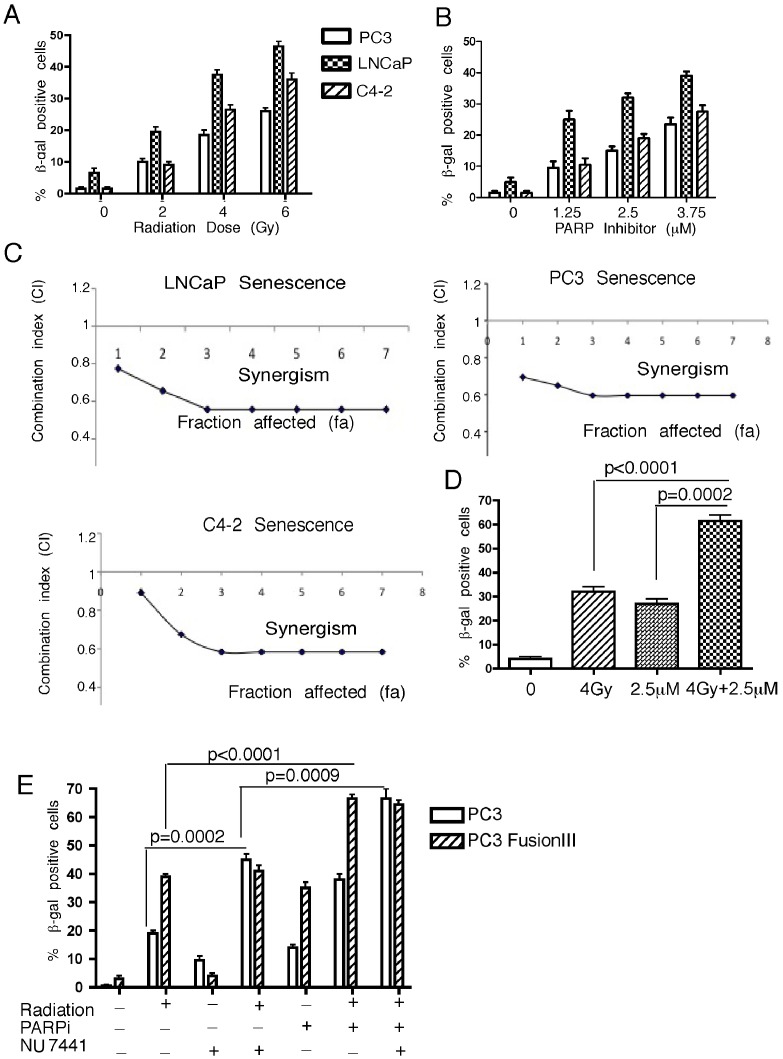
Cellular senescence is activated in *PTEN*-deficient cells. Percentage of β- galactosidase positive cells was determined following radiation (**A**) and rucaparib (**B**),treatment in *PTEN*-deficient LNCaP, C4-2, and PC3 cells. The percentage of β-galactosidase-positive cells was determined by counting ≥five different fields (∼70 cells/sample). **C**, Synergy analyses for senescence in LNCaP, PC3, and C4-2 cells for the combination treatment. See Figs. S2 and 3 for β-galactosidase staining and additional synergy analyses. **D**, Percentage of β-galactosidase positive C4-2 cells was determined after 12 days of treatment with radiation ± rucaparib. **E**, parental and *TMPRSS2-ERG* fusion gene-expressing PC3 cells were quantified for SA-β-galactosidase staining after 6 days following 4 Gy radiation, 2.5 µM rucaparib, and the DNA-PKcs inhibitor NU7441 (500 nM), alone or in combination. Error bars represent SD of mean (n = 3).

Senescence is known to represent an important response to both radiation and PARPi that impacts on cell proliferation and ultimately clonogenic survival. The treated cells showed characteristic markers of senescence after six days. These included flattened cell morphology with the accumulation of SA-β-galactosidase-positive cells (Fig. S3A). *PTEN* null PC3, LNCaP, and C4-2 cell lines showed a radiation and PARPi dose-dependent increase in SA-β-galactosidase-positive cells to treatment either with radiation or rucaparib (Fig. 2A and 2B). LNCaP had the highest number of senescent cells following either single agent or the combination treatment. In contrast, DU145 cells that have a wild-type *PTEN* allele showed almost no senescent cells even at the highest doses used. The combination index for the senescence SA-β-galactosidase staining indicated a moderate-strong synergy (CI = 0.5–0.7) in PC3, LNCaP, and C4-2 cells (Fig. 2C and Fig. S2). The senescence characteristics were sustained up to at least twelve days, with a slight increase in the number of β-galactosidase-positive cells (Fig. 2D and Fig. S3B). These data indicate that senescence contributes to decreased survival of *PTEN*-deficient cells and that the extent of senescence correlates with clonogenic survival.

VCaP cells, which harbor the *TMPRSS2-ERG* fusion gene, acquired senescent cells following radiation and PARPi (Fig. S4). However, these cells are dependent on the fusion gene, therefore examining senescence following knock-down of *TMPRSS2-ERG* was not informative. Hence, we used PC3 cells expressing the same isoform, *TMPRSS2-ERG fusion III* for further senescence experiments. PC3 cells expressing *TMPRSS2-ERG* had an increased number of SA-β-galactosidase-positive cells following radiation (Fig. S4). The effect of *TMPRSS2-ERG* expression was comparable to what was obtained in PC3 cells irradiated in the presence of the DNA-PKcs inhibitor NU7441 (p = 0.0002), while NU7441 alone did not induce senescence in either cell line. Rucaparib treatment in combination with radiation significantly (p<0.0001) increased the number of senescent cells in *TMPRSS2-ERG*-expressing PC3 cells, which again correlated with PC3 cells treated with radiation, rucaparib, and NU7441, (p = 0.0009) (Fig. 2E). NU7441 treatment did not induce senescence in PC3 cells expressing the *TMPRSS2-ERG* fusion following radiation, alone or in combination with rucaparib. These data indicate that DNA-PKcs activity is critical for senescence, as indicated by the increased number of senescent cells in *TMPRSS2-ERG*-expressing cells or when DNA-PKcs is inhibited.

### Rucaparib Increases Persistence of Radiation-induced DNA Damage Foci

**Figure 3:**
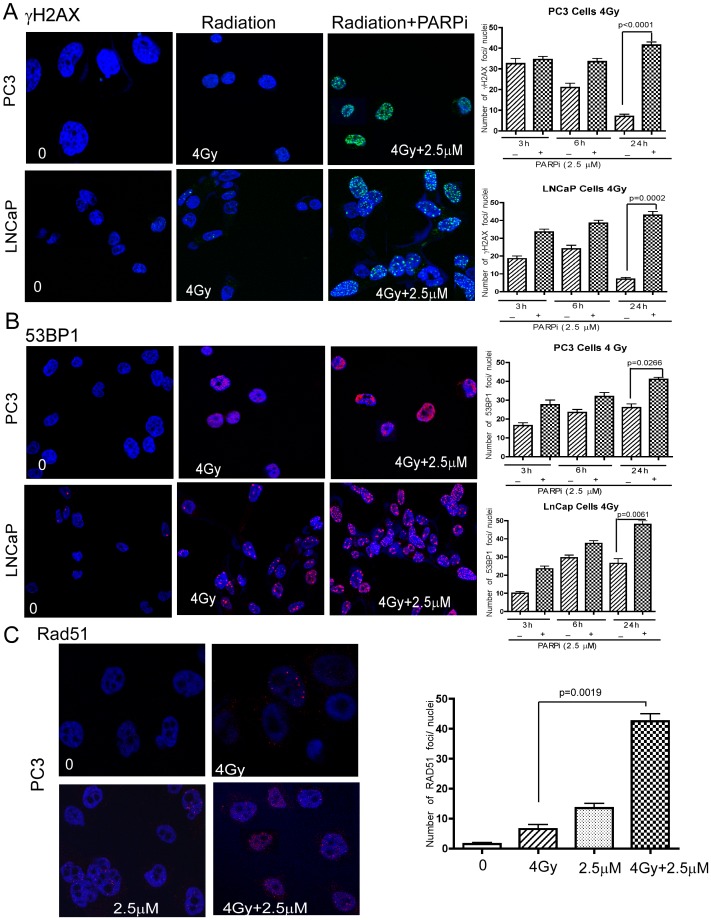
Combination of rucaparib with radiation increases χH2AX and 53BP1 foci leading to persistent DNA damage. **χ**H2AX (**A**) and 53BP1 (**B**) foci were determined by immunofluoresecence microscopy at 24 h following combined treatment with radiation and rucaparib in PC3 and LNCaP cells (left panel), with time-dependent kinetics shown (right panel). **C**, Rad51 foci were visualized and quantified in PC3 cells similarly after 24 h of treatment. Error bars represent SD of mean (n = 3).

We next examined whether the effectiveness of the treatments was related to their ability to induce DNA damage. χH2AX and p53BP1 are established surrogates for measuring ionizing radiation induced foci (IRIF) [24]. Irradiation generated an increased number of χH2AX foci at 3 and 6 h, which were greatly diminished by 24 h, indicative of the repair of the DNA damage (Fig. 3A). In contrast, when cells were irradiated in the presence of rucaparib, χH2AX foci persisted at 24 h. In addition, foci for p53BP1, another established marker for DSBs, were more prominent at 24 h following irradiation, with the combined treatment with rucaparib resulting in an increased number of foci (Fig. 3B). Rad51 is a key component of HR; its expression was not affected by the *PTEN* status, consistent with a recent report [25]. Nevertheless, the combination of rucaparib and radiation showed persistent Rad51 foci at 24 h (Fig. 3C), indicating that the combination is more effective in inflicting persistent DNA damage in the treated cells.

### LDR Leads to Increased DNA Damage and Reduced Cell Survival

**Figure 4:**
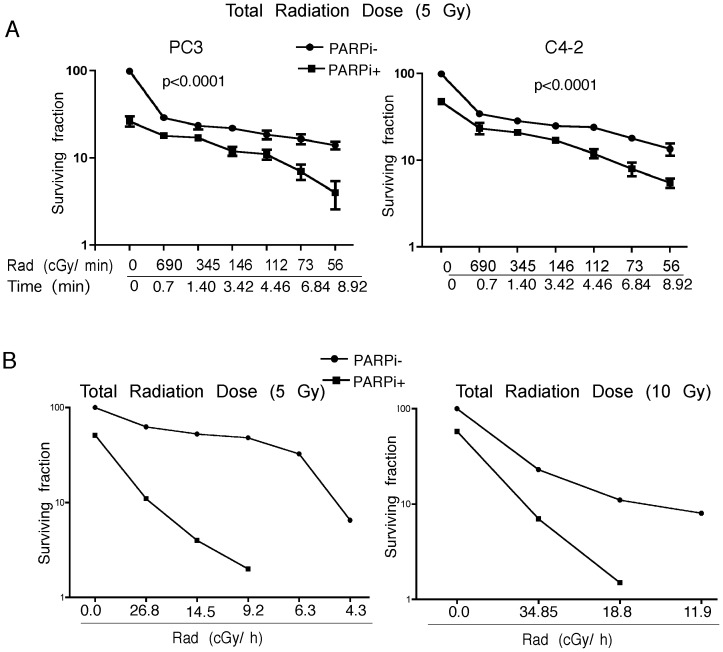
LDR is effective in sensitizing PCa Cells. **A**, Clonogenic survival assays were performed for PC3 (left panel) and C4-2 cells (right panel) at different dose-rates ±1.25 µM rucaparib. **B**, C4-2 cells were exposed to different dose rates of radiation from an Ir-192 source ±2.5 µM rucaparib. A total radiation dose of 5 Gy (left panel) and 10 Gy (right panel) was delivered; therefore the time of exposure differed for the various dose-rates used. Error bars represent SD of mean (n = 3).

**Figure 5:**
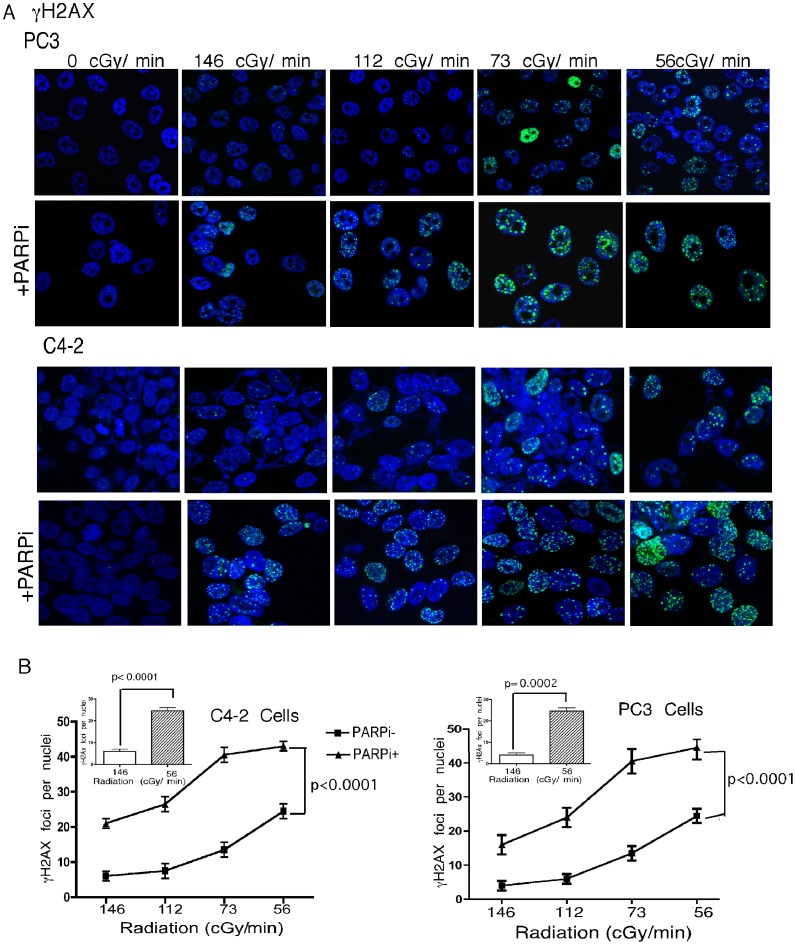
LDR induces enhanced DNA damage in the presence of rucaparib. **A**, Confocal immunostaining for χH2AX foci, enumarated in PC3 and C4-2 cells following radiation at the indicated dose rates. **B**, Quantitation of dose-dependent formation of γH2AX foci. Error bars represent SD of mean (n = 3).

**Figure 6:**
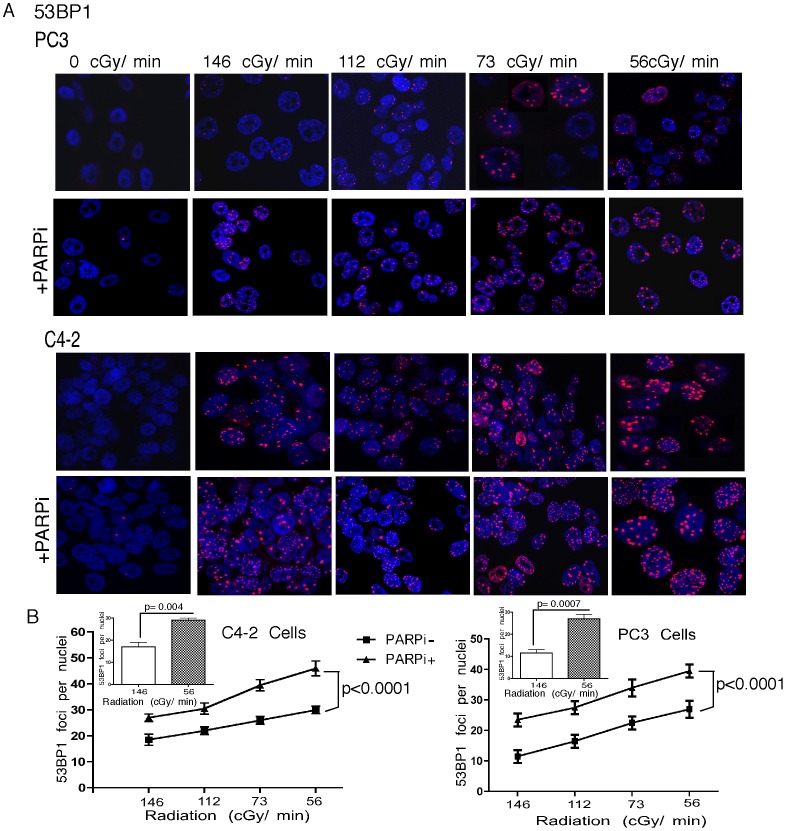
LDR, when combined with rucaparib, augments DNA damage-induced 53BP1 IRIF. **A**, Confocal immunostaining of 53BP1 foci in PC3 and C4-2 cells following radiation at the indicated dose rates. **B**, Graphical representation of dose-dependent 53BP1 IRIF generation. Error bars represent SD of mean (n = 3).

Radiation is administered in the clinic either as external beam radiotherapy (EBRT) or brachytherapy. Radiation for the treatment of PCa by brachytherapy typically utilizes dose rates of <70 cGy/h as compared to 200–300 cGy/min that are commonly used for EBRT for *in vitro* radiation studies of human cancer cell lines with conventional irradiators. To investigate the effectiveness of lower dose-rates, C4-2 and PC3 cells were exposed to dose rates of 56 to 690 cGy/min, to achieve a total dose of 5Gy. The longer exposure time directly correlated with more extensive DNA damage, resulting in fewer colonies (Fig. 4A) and more χH2AX and 53BP1 foci (Fig. 5A and 6A). The lowest dose rate (56 cGy/min) increased the number of χH2AX foci significantly (p<0.0002) compared to the conventional dose rate (146 cGy/min). Addition of rucaparib significantly (p<0.0001) induced DNA damage as measured by an increased number of χH2AX foci (Fig. 5B). Moreover, there were significantly (p<0.0001) increased numbers of p53BP1 foci following the combination treatment (Fig. 6B). The lowest dose rate of radiation (56 cGy/min) induced significantly more 53BP1 IRIF (p = 0.004 & 0.0007 for C4-2 and PC3 cells respectively) compared to the highest dose rate (690 cGy/min).

Similar experiments were performed with an lr-192 radiation source (fixed total dose of 5 and 10 Gy) ± rucaparib. The cells which received the lowest dose-rate (4.34 cGy/h), delivered over the longest period of time, formed fewer colonies compared to those exposed to moderate to higher doses (26.8 cGy/h) of radiation (Fig. 4B). Rucaparib greatly reduced colony formation even at the highest LDR dose tested (26.8 cGy/h), which required ∼18 h to achieve 5 Gy.

### PARP Inhibition Radiosensitizes TMPRSS2-ERG Fusion Gene-expressing Cells to an Extent Similar to DNA-PKcs Inhibition in Parental Cells

**Figure 7:**
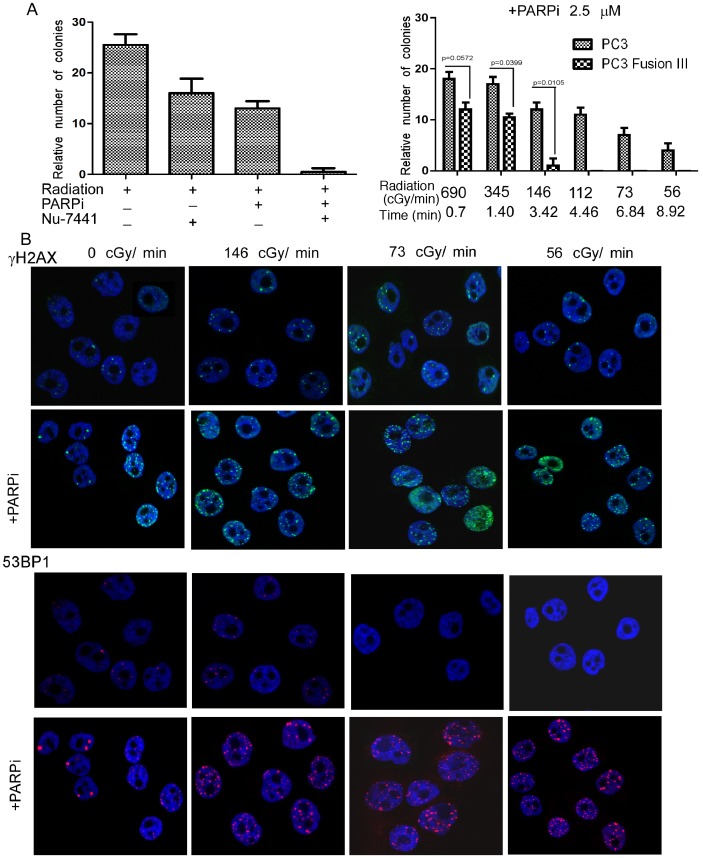
The *TMPRSS2-ERG* fusion gene provides enhanced sensitivity to PARP inhibition, comparable to that of DNA-PKcs inhibition in parental cells. A, Clonogenic survival assay in PC3 cells following 4 Gy radiation ±2.5 µM rucaparib and the DNA-PKCs inhibitor NU7441 (500 nM; left panel). B, Clonogenic survival assays in PC3 cells and derivatives expressing TMPRSS2-ERG fusion III at different dose-rates ±2.5 µM rucaparib. C, Immunostaining for χH2AX and 53BP1 in PC3 cells expressing the fusion gene following radiation ± rucaparib treatment for 24 h. Error bars represent SD of mean (n = 3).

The fusion between *TMPRSS2*, an androgen-regulated oncogene, and an ETS transcription factor estrogen-regulated gene, ERG generated by an interstitial deletion on chromosome 21 or by reciprocal translocation is present in ∼50% of early stage PCa [26]. VCaP cells that express *TMPRSS2-ERG* endogenously were radiosensitized by rucaparib (Fig. 1B and Fig. S2A). These cells are dependent on *TMPRSS2-ERG* as they stop proliferating when it is depleted by siRNA (data not shown). Therefore, PC3 cells were transfected with the *TMPRSS2-ERG* fusion III isoform, the most common PCa fusion gene. Its stable expression did not have a significant effect on radiosensitivity, estimated by clonogenic survival assays (Fig. S1B) and by χH2AX and 53BP1 foci. However, when it was administered together with rucaparib, the number of colonies was reduced significantly (p = 0.0105) (Fig. 7B). This effect was comparable to what was obtained in PC3 cells treated with the DNA-PKcs inhibitor NU7441. The rucaparib combination further radiosensitized these cells (p = 0.0005), to an extent comparable to cells expressing the *TMPRSS2-ERG* fusion gene treated with rucaparib (Fig. 7A). An increased number of 53BP1 and χH2AX foci were visible even at the lowest dose rate, further demonstrating that rucaparib is a potent radiosensitizer for PCa cells that harbor a fusion gene (Fig. 7C).

## Discussion

Rucaparib (CO-338; formerly known as AG014699 and PF-01367338), a PARPi represents a novel class of agents with documented antitumor activity against human cell lines and xenografts containing mutated or epigenetically silenced BRCA1/2 [4], [27]. Prior work has established that PARP-1 interacts with TRMPRSS2-ERG, providing thus a mechanistic rationale for the use of PARPi in ETS gene fusion-positive PCa [3]. Here we show for the first time its effectiveness as a radiation sensitizer in a panel of PCa cell lines, with maximal synergy achieved with low dose-rate (LDR) rather than conventional dose radiation. The LDR doses used here mimick those employed in clinical practice for PCa brachythrepay [28]. We believe this finding is important since brachytherapy is more effective than EBRT for PCa treatment, and it may employ molecular mechanisms that promote enhanced cancer cell radiosensitivity, in our study through enhanced senescence. Indeed, chemical inhibition of PARP-1 activity induced marked radiosensitization of several exponentially growing tumor cell lines in the 5–30 cGy dose range. LDR radiosensitization of actively dividing tumor cells by PARPi suggests that they may have a role in enhancing the efficacy of ultra-fractionated or LDR regimens [29]. Our studies indicate that rucaparib is a very potent PARPi that synergizes with clinical doses of radiation achieved during brachytherapy. Its use as a rediosensitizer is effective even when the radiation dose is greatly reduced, a situation encountered as the radioactive “seeds” decay during the extensive time they are used in PCa patients.

Rucaparib, the first PARPi that was developed and tested in the clinic (in 2003 under the name AG014699) [22], [23], has been also shown to be effective in tumor xenografts of breast, lung, colon, colorectal, and pancreatic cancer [27], [30]. At the same time, it has been shown to be nontoxic in mice that carried at least one functional copy of the BRCA2 gene. Our study is the first ever carried out for rucaparib in PCa. While we have not pursued similar xenograft studies, based on the above reports with diverse tumor types, all indications are that these results would be translated to PCa xenograft models.

Rucaparib has been examined in combination with various chemotherapeutic agents. It was found to be effective in combination with platinum in breast and ovarian cancer xenograft models [27], [31]. Our study is the first one to examine its effectiveness in combination with radiotherapy. Other PARPi, in combination with irradiation, were reported to cause significant tumor growth delay in lung cancer *in vivo* models [8], and to be effective radiosensitizers in both lung and PCa cell lines [32]. Sensitization to radiation and alkylating agents was enhanced in DSB repair-deficient cells [33], consistent with the remarkable sensitivity to PARP inhibition of BRCA-1 and BRCA-2-deficient tumor cells [6]. The ABT-888 (veliparib) PARPi was recently shown to enhance the response of PCa cells and tumors to irradiation in DU145 and PC3 cells [34]. Combining ABT-888 with 6 Gy resulted in delayed tumor regrowth compared with either agent alone only in PC3 xenograft tumors, whereas DU145 tumors continued to grow. Similar to our studies, PC3 but not DU145 cells and tumors were shown to contain abundant senescent cells displaying persistent DNA damage foci [34]. We show that rucaparib is effective in additional *PTEN*-deficient cells including those that harbor ETS gene fusions. Clearly, the efficacy of PARPi may depend on a competent senescence response to accumulated DNA damage, such as when *PTEN* is deficient or when the *TMPRSS2-ERG* is expressed. A recent study has found that rucaparib radiozensitization can be also NF-κB dependent [35]. In this study we show that *TMPRSS2-ERG*, in addition to *PTEN* deficiency, can sensitize to PARPi, which synergizes with radiotherapy.

Since mutations in BRCA1/2 that provide a “*synthetic lethality*” relationship with PARPi are rare in PCa, there is considerable interest in defining other molecular markers for PARPi sensitivity. Indeed, sensitization to PARPi has been recently shown to be augmented by blocking DNA damage signaling, through ATM/Chk2 [36], [37] and the MRN complex through Mre11 [38]. In contrast, blocking NHEJ DNA repair through p53BP1 [39], can in fact counteract the exquisite sensitivity to PARPi caused by BRCA1 deficiency [40]. On the other hand, expression of p53 in our PCa cell panel and a recent report [39] did not make a difference as both p53 null (PC3) or p53 proficient (LNCaP, C4-2) cells responded as well to PARPi as a radiosensitizer. The response was rather dependent primarily on a competent senescent response that was absent in DU145 cells that have a functional *PTEN* allele and a truncated, non-functional retinoblastoma tumor suppressor [41].

ETS gene fusions are found in the majority of PCa cases [26]. The expression of the fusion gene is responsible for cell proliferation in PCa cells that express the *TMPRSS2-ERG* [42]. Mouse prostates devoid of *PTEN* display enhanced tumor genesis in the presence of overexpressed ERG [43]. A recent report showed that fusion gene expression alters radio- and chemo-sensitivity when X-ray radiation is administered in combination with paclitaxel [44]. Similar to BRCA1/2 deficiency, inhibitors of PARP-1 enhance the extent of DNA damage promoted initially by overexpression of fusion genes [3]. Therefore, prostate tumor cells harboring ETS fusions, such as *TMPRSS2-ERG* are more prone to robust response to PARPi, alone on in combination with LDR. Another study has, however, found that *PTEN* deletion in PCa cells may not necessarily associate with loss of RAD51 function [25], with sensitivity to a different PARPi being associated instead with a defect in MRE11 expression. We discovered that the DNA-PKcs inhibition by *TMPRSS2-ERG (data not shown)* has a critical role for senescence activation. Remarkably, DNA-PKcs expression has been recently shown to predict the response to radiotherapy in PCa [45].

In addition to its role in DNA damage and repair, a transcriptional role has been also attributed to PARP-1 [3], most recently indicating that it is required for androgen receptor function, particularly in castration-resistant models of PCa [46]. As we have shown earlier, *TMPRSS2-ERG* expression can impact on androgen receptor signaling, for example by decreasing levels of critical prostate-relevant markers, such as the prostate specific membrane antigen, PSMA [47].

In summary, our studies show a synergistic interaction of a potent PARPi, rucaparib, in PCa tumor cells when it is combined with radiation. LDR radiation mimicking brachytherapy was more effective than a radiation dose equivalent to what is used clinically for EBRT. Synergy achieved for clonogenic survival correlated with that for senescence in *PTEN*-deficient cells and cells expressing the *TMPRSS2-ERG* fusion gene. These data support the effectiveness of rucaparib as a potent PARPi for radiosensitizing PCa cells, particularly those that express the *TMPRSS2-ERG* gene fusion and are *PTEN*-deficient, indicating potential clinical application for brachytherapy in patients with intermediate and high risk PCa.

## Supporting Information

Figure S1 **Staining for SA-β-galactosidase.** **A**: Staining after 6 days revealed β-galactosidase-positive LNCaP, C4-2, and PC3 but not DU145 cells, which harbor a wild-type PTEN allele, following treatment with radiation and rucaparib, alone or in combination. **B**: Similar experiments were carried out for a 12-day β-galactosidase staining.
(TIF)

Figure S2 **Synergistic effect of combination of radiation and rucaparib on clonogenic survival and senescence.** The combination index and fraction affected (fa) were estimated based on (**A**:) clonogenic survival assay and (**B**:) β-galatosidase staining as a proxy for senescence. CI values <1 represent synergy, CI > 1 an antagonistic interaction between the two treatments.
(TIF)

Figure S3 **Expression of the TMPRSS2-ERG fusion III isoform.** Western blot indicates stable V5-tagged fusion III isoform expression in PC3 cells using antibodies against V5 and β-actin, as a loading control.
(TIF)

Figure S4 **Positive Staining of SA-β-galactosidase.** **A**, VCaP cells with or without TMPRSS2-ERG fusion (siERG) exhibit positively stained cells following radiation and rucaparib alone or in combination. **B**, PC3 cells expressing TMPRSS2-ERG fusion gene display SA-β-galactoside staining following radiation ± rucaparib.
(TIF)

## References

[1] Siegel R, Naishadham D, Jemal A (2012) Cancer statistics, 2012. CA Cancer J Clin 62: 10–29. 22237781 10.3322/caac.20138

[2] Rouleau M, Patel A, Hendzel MJ, Kaufmann SH, Poirier GG (2010) PARP inhibition: PARP1 and beyond. Nat Rev Cancer 10: 293–301. 20200537 10.1038/nrc2812 PMC2910902

[3] Brenner JC, Ateeq B, Li Y, Yocum AK, Cao Q, et al (2011) Mechanistic rationale for inhibition of poly(ADP-ribose) polymerase in ETS gene fusion-positive prostate cancer. Cancer Cell 19: 664–678. 21575865 10.1016/j.ccr.2011.04.010 PMC3113473

[4] Javle M, Curtin NJ (2011) The role of PARP in DNA repair and its therapeutic exploitation. Br J Cancer 105: 1114–1122. 21989215 10.1038/bjc.2011.382 PMC3208503

[5] Sugimura K, Takebayashi S, Taguchi H, Takeda S, Okumura K (2008) PARP-1 ensures regulation of replication fork progression by homologous recombination on damaged DNA. J Cell Biol 183: 1203–1212. 19103807 10.1083/jcb.200806068 PMC2606964

[6] Bryant HE, Schultz N, Thomas HD, Parker KM, Flower D, et al (2005) Specific killing of BRCA2-deficient tumours with inhibitors of poly(ADP-ribose) polymerase. Nature 434: 913–917. 15829966 10.1038/nature03443

[7] Patel AG, Sarkaria JN, Kaufmann SH (2011) Nonhomologous end joining drives poly(ADP-ribose) polymerase (PARP) inhibitor lethality in homologous recombination-deficient cells. Proc Natl Acad Sci U S A 108: 3406–3411. 21300883 10.1073/pnas.1013715108 PMC3044391

[8] Albert JM, Cao C, Kim KW, Willey CD, Geng L, et al (2007) Inhibition of poly(ADP-ribose) polymerase enhances cell death and improves tumor growth delay in irradiated lung cancer models. Clin Cancer Res 13: 3033–3042. 17505006 10.1158/1078-0432.CCR-06-2872

[9] Schiewer MJ, Goodwin JF, Han S, Brenner JC, Augello MA, et al. (2012) Dual roles of PARP-1 promote cancer growth and progression. Cancer Discov. 10.1158/2159-8290.CD-12-0120 PMC3519969 22993403

[10] Zaremba T, Thomas HD, Cole M, Coulthard SA, Plummer ER, et al (2011) Poly(ADP-ribose) polymerase-1 (PARP-1) pharmacogenetics, activity and expression analysis in cancer patients and healthy volunteers. Biochem J 436: 671–679. 21434873 10.1042/BJ20101723

[11] Lord CJ, Ashworth A (2012) The DNA damage response and cancer therapy. Nature 481: 287–294. 22258607 10.1038/nature10760

[12] Salmena L, Carracedo A, Pandolfi PP (2008) Tenets of PTEN tumor suppression. Cell 133: 403–414. 18455982 10.1016/j.cell.2008.04.013

[13] DuPree EL, Mazumder S, Almasan A (2004) Genotoxic stress induces expression of E2F4, leading to its association with p130 in prostate carcinoma cells. Cancer Res 64: 4390–4393. 15231644 10.1158/0008-5472.CAN-03-3695

[14] Wang J, Cai Y, Yu W, Ren C, Spencer DM, et al (2008) Pleiotropic biological activities of alternatively spliced TMPRSS2/ERG fusion gene transcripts. Cancer Res 68: 8516–8524. 18922926 10.1158/0008-5472.CAN-08-1147 PMC2597580

[15] Mazumder S, Plesca D, Kintner M, Almasan A (2007) Interaction of a Cyclin E fragment with Ku70 regulates Bax-mediated apoptosis in hematopoietic cells. Mol Cell Biol 27: 3511–3520. 17325036 10.1128/MCB.01448-06 PMC1899959

[16] Kunos CA, Colussi VC, Pink J, Radivoyevitch T, Oleinick NL (2011) Radiosensitization of human cervical cancer cells by inhibiting ribonucleotide reductase: enhanced radiation response at low-dose rates. Int J Radiat Oncol Biol Phys 80: 1198–1204. 21470790 10.1016/j.ijrobp.2011.01.034 PMC3118909

[17] Singh K, Matsuyama S, Drazba JA, Almasan A (2012) Autophagy-dependent senescence in response to DNA damage and chronic apoptotic stress. Autophagy 8: 236 – 251. 22240589 10.4161/auto.8.2.18600 PMC3336077

[18] Ray S, Bucur O, Almasan A (2005) Sensitization of prostate carcinoma cells to Apo2L/TRAIL by a Bcl-2 family protein inhibitor. Apoptosis 10: 1411–1418. 16215673 10.1007/s10495-005-2490-y

[19] Ray S, Shyam S, Fraizer GC, Almasan A (2007) S-phase checkpoints regulate Apo2 ligand/TRAIL and CPT-11-induced apoptosis of prostate cancer cells. Mol Cancer Ther 6: 1368–1378. 17431115 10.1158/1535-7163.MCT-05-0414

[20] Crosby ME, Jacobberger J, Gupta D, Macklis RM, Almasan A (2007) E2F4 regulates a stable G2 arrest response to genotoxic stress in prostate carcinoma. Oncogene 26: 1897–1909. 17043659 10.1038/sj.onc.1209998 PMC2593901

[21] Fong PC, Boss DS, Yap TA, Tutt A, Wu P, et al (2009) Inhibition of poly(ADP-ribose) polymerase in tumors from BRCA mutation carriers. N Engl J Med 361: 123–134. 19553641 10.1056/NEJMoa0900212

[22] Plummer R, Jones C, Middleton M, Wilson R, Evans J, et al (2008) Phase I study of the poly(ADP-ribose) polymerase inhibitor, AG014699, in combination with temozolomide in patients with advanced solid tumors. Clin Cancer Res 14: 7917–7923. 19047122 10.1158/1078-0432.CCR-08-1223 PMC2652879

[23] Curtin NJ (2012) DNA repair dysregulation from cancer driver to therapeutic target. Nat Rev Cancer 12: 801–817. 23175119 10.1038/nrc3399

[24] Sharma A, Singh K, Almasan A (2012) Histone H2AX phosphorylation: A marker for DNA damage. Methods Mol Biol 920: 613–626. 22941631 10.1007/978-1-61779-998-3_40

[25] Fraser M, Zhao H, Luoto KR, Lundin C, Coackley CL, et al (2012) PTEN deletion in prostate cancer cells does not associate with loss of RAD51 function: implications for radiotherapy and chemotherapy. Clin Cancer Res 18 1015–1027. 22114138 10.1158/1078-0432.CCR-11-2189 PMC3378487

[26] Rubin MA, Maher CA, Chinnaiyan AM (2011) Common gene rearrangements in prostate cancer. J Clin Oncol 29: 3659–3668. 21859993 10.1200/JCO.2011.35.1916 PMC4874145

[27] Drew Y, Mulligan EA, Vong WT, Thomas HD, Kahn S, et al (2011) Therapeutic potential of poly(ADP-ribose) polymerase inhibitor AG014699 in human cancers with mutated or methylated BRCA1 or BRCA2. J Natl Cancer Inst 103: 334–346. 21183737 10.1093/jnci/djq509

[28] Nag S (2000) Brachytherapy for prostate cancer: summary of American Brachytherapy Society recommendations. Semin Urol Oncol 18: 133–136. 10875454

[29] Chalmers A, Johnston P, Woodcock M, Joiner M, Marples B (2004) PARP-1, PARP-2, and the cellular response to low doses of ionizing radiation. Int J Radiat Oncol Biol Phys 58: 410–419. 14751510 10.1016/j.ijrobp.2003.09.053

[30] Ali M, Kamjoo M, Thomas HD, Kyle S, Pavlovska I, et al (2011) The clinically active PARP inhibitor AG014699 ameliorates cardiotoxicity but doesn't enhance the efficacy of doxorubicin, despite improving tumor perfusion and radiation response in mice. Mol Cancer Ther 10: 2320–9. 21926192 10.1158/1535-7163.MCT-11-0356 PMC3242069

[31] Mukhopadhyay A, Curtin N, Plummer R, Edmondson RJ (2011) PARP inhibitors and epithelial ovarian cancer: an approach to targeted chemotherapy and personalised medicine. BJOG 118: 429–432. 21244617 10.1111/j.1471-0528.2010.02838.x

[32] Liu SK, Coackley C, Krause M, Jalali F, Chan N, et al (2008) A novel poly(ADP-ribose) polymerase inhibitor, ABT-888, radiosensitizes malignant human cell lines under hypoxia. Radiother Oncol 88: 258–268. 18456354 10.1016/j.radonc.2008.04.005

[33] Loser DA, Shibata A, Shibata AK, Woodbine LJ, Jeggo PA, et al (2010) Sensitization to radiation and alkylating agents by inhibitors of poly(ADP-ribose) polymerase is enhanced in cells deficient in DNA double-strand break repair. Mol Cancer Ther 9: 1775–1787. 20530711 10.1158/1535-7163.MCT-09-1027 PMC2884153

[34] Barreto-Andrade JC, Efimova EV, Mauceri HJ, Beckett MA, Sutton HG, et al (2011) Response of human prostate cancer cells and tumors to combining PARP inhibition with ionizing radiation. Mol Cancer Ther 10: 1185–1193. 21571912 10.1158/1535-7163.MCT-11-0061 PMC3140695

[35] Hunter JE, Willmore E, Irving JA, Hostomsky Z, Veuger SJ, et al. (2011) NF-kappaB mediates radio-sensitization by the PARP-1 inhibitor, AG-014699. Oncogene. 10.1038/onc.2011.229 PMC3191117 21706052

[36] Williamson CT, Muzik H, Turhan AG, Zamo A, O′Connor MJ, et al (2010) ATM deficiency sensitizes mantle cell lymphoma cells to poly(ADP-ribose) polymerase-1 inhibitors. Mol Cancer Ther 9: 347–357. 20124459 10.1158/1535-7163.MCT-09-0872 PMC3729269

[37] Hoglund A, Stromvall K, Li Y, Forshell LP, Nilsson JA (2011) Chk2 deficiency in Myc overexpressing lymphoma cells elicits a synergistic lethal response in combination with PARP inhibition. Cell Cycle 10: 3598–3607. 22030621 10.4161/cc.10.20.17887 PMC3266184

[38] Vilar E, Bartnik CM, Stenzel SL, Raskin L, Ahn J, et al (2011) MRE11 deficiency increases sensitivity to poly(ADP-ribose) polymerase inhibition in microsatellite unstable colorectal cancers. Cancer Res 71: 2632–2642. 21300766 10.1158/0008-5472.CAN-10-1120 PMC3407272

[39] Oplustilova L, Wolanin K, Mistrik M, Korinkova G, Simkova D, et al (2012) Evaluation of candidate biomarkers to predict cancer cell sensitivity or resistance to PARP-1 inhibitor treatment. Cell Cycle 11: 3837–3850. 22983061 10.4161/cc.22026 PMC3495826

[40] Bunting SF, Callen E, Wong N, Chen HT, Polato F, et al (2010) 53BP1 inhibits homologous recombination in Brca1-deficient cells by blocking resection of DNA breaks. Cell 141: 243–254. 20362325 10.1016/j.cell.2010.03.012 PMC2857570

[41] Bookstein R, Shew JY, Chen PL, Scully P, Lee WH (1990) Suppression of tumorigenicity of human prostate carcinoma cells by replacing a mutated RB gene. Science 247: 712–715. 2300823 10.1126/science.2300823

[42] Sun C, Dobi A, Mohamed A, Li H, Thangapazham RL, et al (2008) TMPRSS2-ERG fusion, a common genomic alteration in prostate cancer activates C-MYC and abrogates prostate epithelial differentiation. Oncogene 27: 5348–5353. 18542058 10.1038/onc.2008.183 PMC7556723

[43] Carver BS, Tran J, Gopalan A, Chen Z, Shaikh S, et al (2009) Aberrant ERG expression cooperates with loss of PTEN to promote cancer progression in the prostate. Nat Genet 41: 619–624. 19396168 10.1038/ng.370 PMC2835150

[44] Swanson TA, Krueger SA, Galoforo S, Thibodeau BJ, Martinez AA, et al. (2011) TMPRSS2/ERG fusion gene expression alters chemo- and radio-responsiveness in cell culture models of androgen independent prostate cancer. Prostate: e-pub. 10.1002/pros.21371 21394739

[45] Bouchaert P, Guerif S, Debiais C, Irani J, Fromont G (2012) DNA-PKcs Expression Predicts Response to Radiotherapy in Prostate Cancer. Int J Radiat Oncol Biol Phys. 84: 1179–85. 10.1016/j.ijrobp.2012.02.014 22494583

[46] Schiewer MJ, Goodwin JF, Han S, Brenner JC, Augello MA, et al (2012) Dual Roles of PARP-1 Promote Cancer Growth and Progression. Cancer Discov 2: 1134–1149. 22993403 10.1158/2159-8290.CD-12-0120 PMC3519969

[47] Yin L, Rao P, Elson P, Wang J, Ittmann M, et al (2011) Role of TMPRSS2-ERG gene fusion in negative regulation of PSMA expression. PLoS One 6: e21319. 21731703 10.1371/journal.pone.0021319 PMC3123299

