# Supplementary figures and images for: PARP Inhibition Sensitizes to Low Dose-Rate Radiation TMPRSS2-ERG Fusion Gene-Expressing and PTEN-Deficient Prostate Cancer Cells
Source: PLoS One. 2013 Apr 2;8(4):e60408. doi: 10.1371/journal.pone.0060408 (PMC3614551; doi:10.1371/journal.pone.0060408)

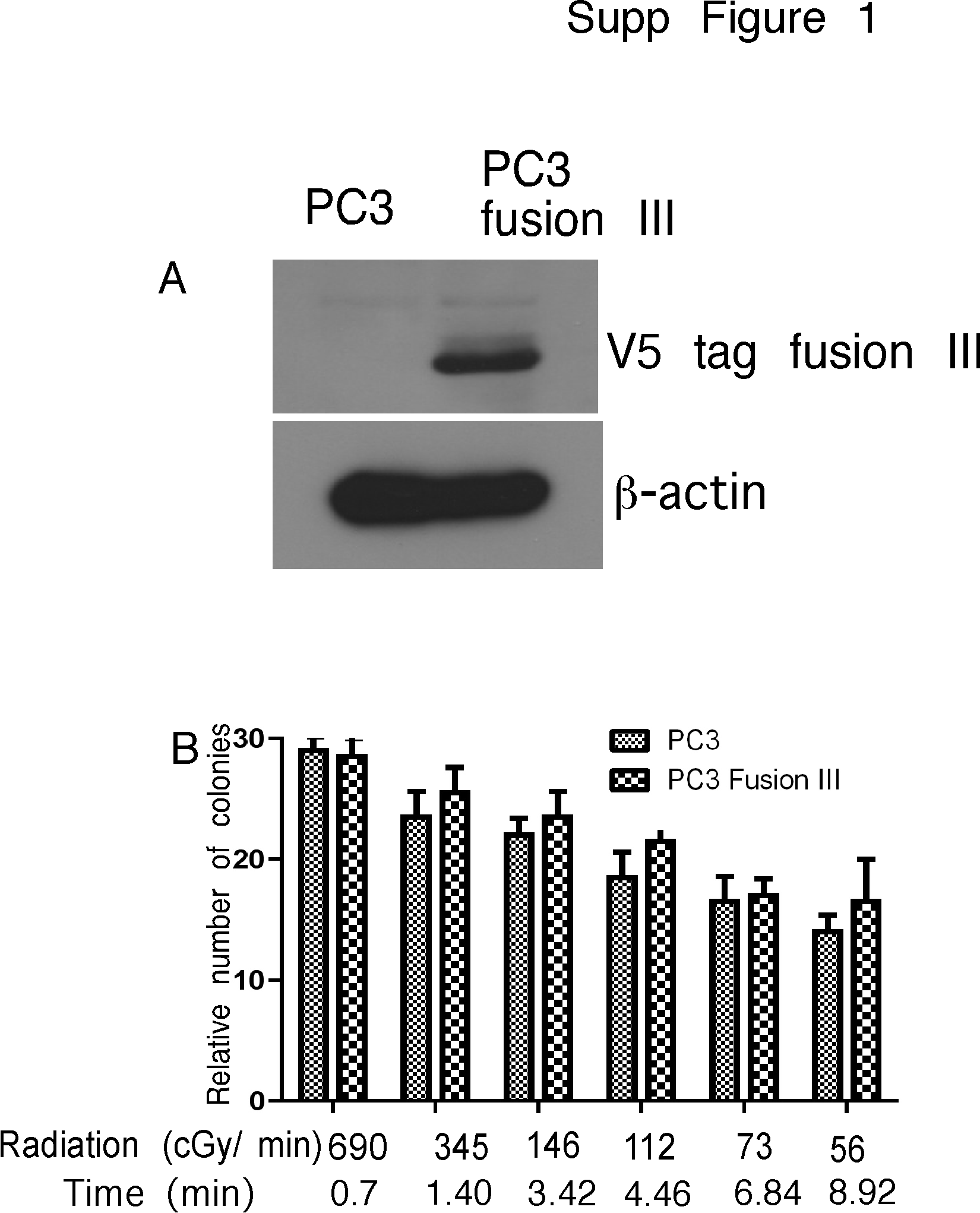

Supplement: Figure S1 — Staining for SA-β-galactosidase. A: Staining after 6 days revealed β-galactosidase-positive LNCaP, C4-2, and PC3 but not DU145 cells, which harbor a wild-type PTEN allele, following treatment with radiation and rucaparib, alone or in combination. B: Similar experiments were carried out for a 12-day β-galactosidase staining. (TIF) [file pone.0060408.s001.tif]

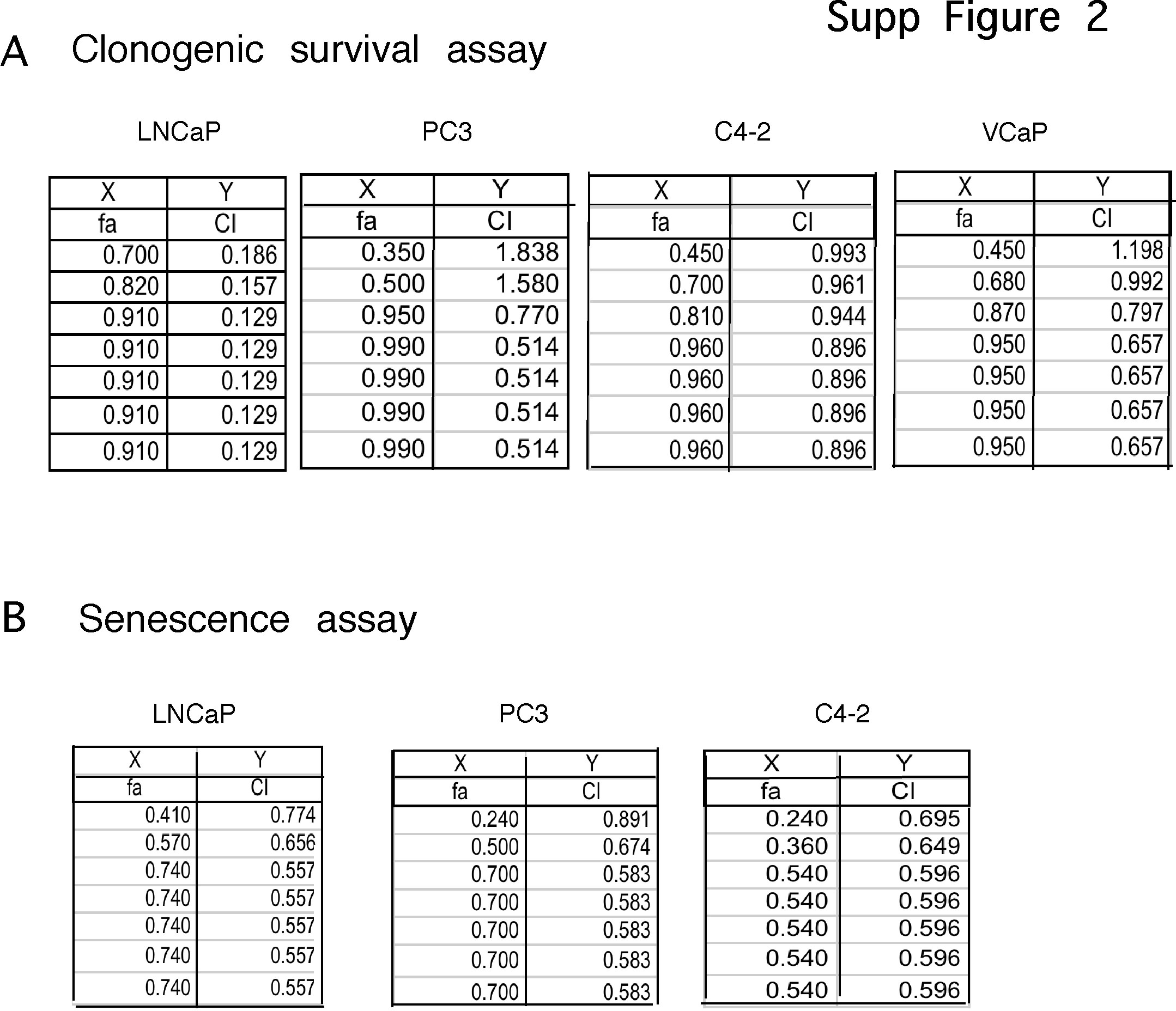

Supplement: Figure S2 — Synergistic effect of combination of radiation and rucaparib on clonogenic survival and senescence. The combination index and fraction affected (fa) were estimated based on (A:) clonogenic survival assay and (B:) β-galatosidase staining as a proxy for senescence. CI values <1 represent synergy, CI > 1 an antagonistic interaction between the two treatments. (TIF) [file pone.0060408.s002.tif]

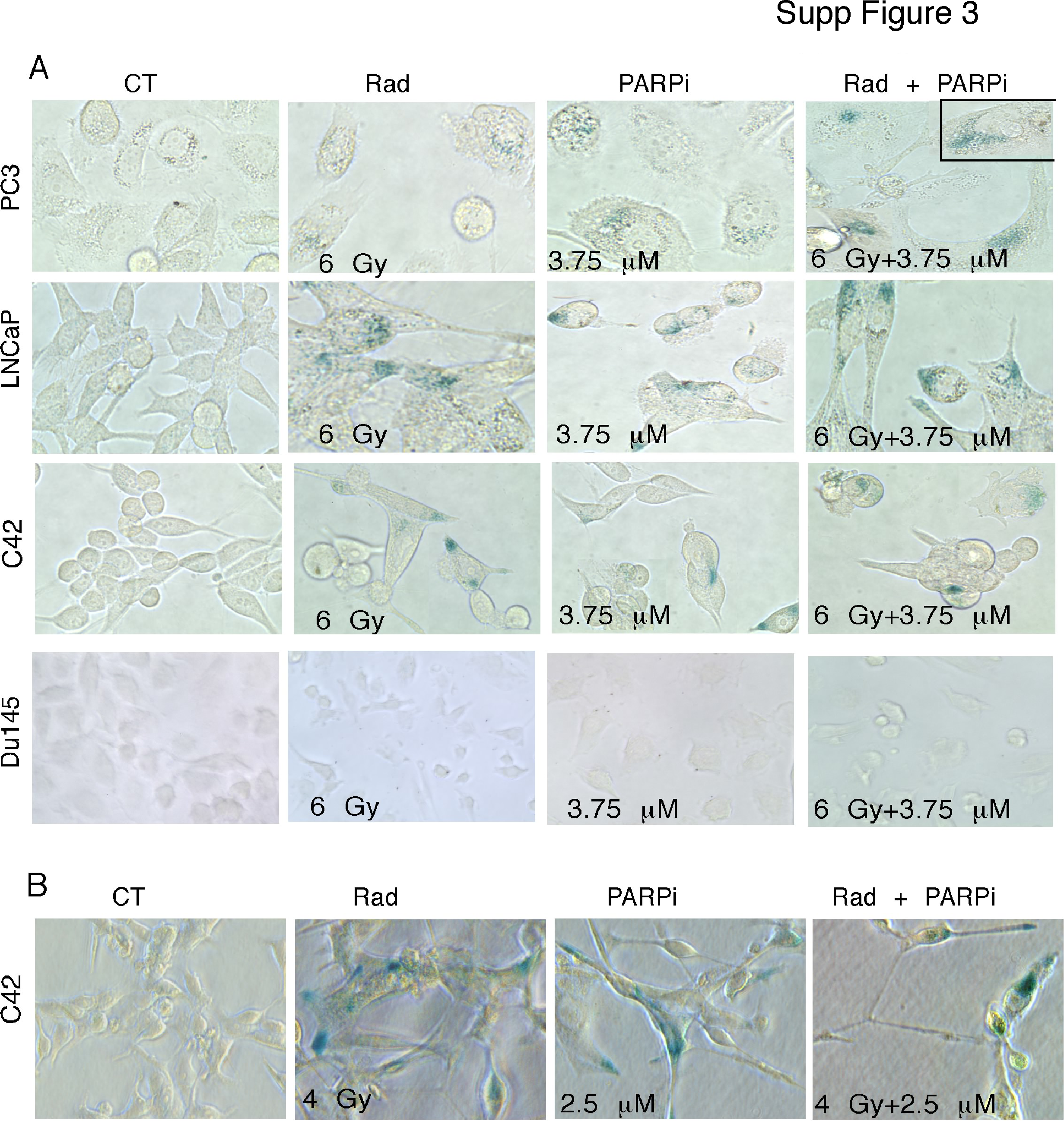

Supplement: Figure S3 — Expression of the TMPRSS2-ERG fusion III isoform. Western blot indicates stable V5-tagged fusion III isoform expression in PC3 cells using antibodies against V5 and β-actin, as a loading control. (TIF) [file pone.0060408.s003.tif]

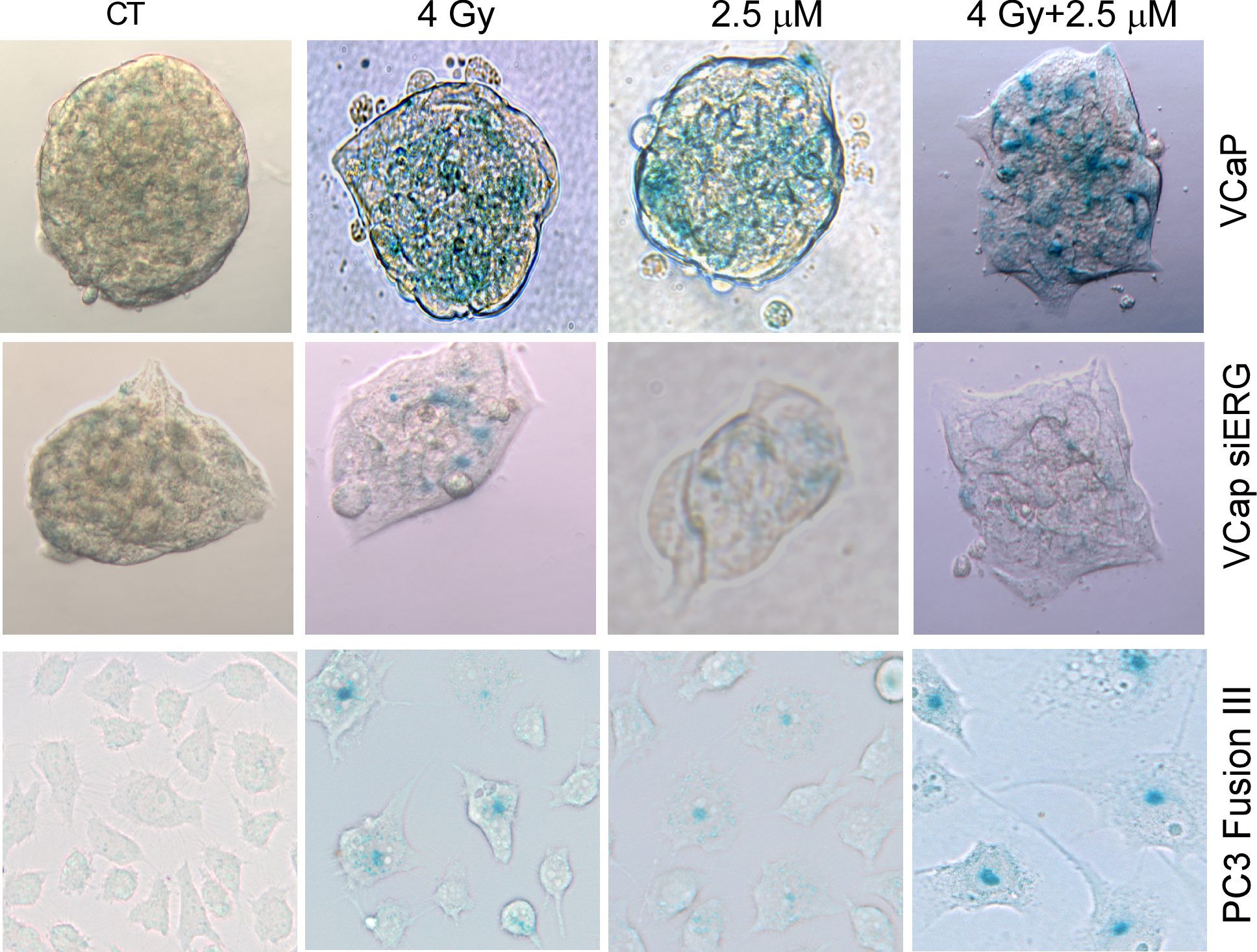

Supplement: Figure S4 — Positive Staining of SA-β-galactosidase. A, VCaP cells with or without TMPRSS2-ERG fusion (siERG) exhibit positively stained cells following radiation and rucaparib alone or in combination. B, PC3 cells expressing TMPRSS2-ERG fusion gene display SA-β-galactoside staining following radiation ± rucaparib. (TIF) [file pone.0060408.s004.tif]
